# Publisher Correction: Free-standing two-dimensional ferro-ionic memristor

**DOI:** 10.1038/s41467-024-53167-8

**Published:** 2024-11-07

**Authors:** Jinhyoung Lee, Gunhoo Woo, Jinill Cho, Sihoon Son, Hyelim Shin, Hyunho Seok, Min-Jae Kim, Eungchul Kim, Ziyang Wang, Boseok Kang, Won-Jun Jang, Taesung Kim

**Affiliations:** 1 School of Mechanical Engineering, Sungkyunkwan University (SKKU), Suwon-si, Gyeonggi-do 16419 Republic of Korea; 2 Center for Quantum Nanoscience, Institute for Basic Science (IBS), Seoul, 03760 Republic of Korea; 3 SKKU Advanced Institute of Nanotechnology (SAINT), Sungkyunkwan University, Suwon-si, Gyeonggi-do 16419 Republic of Korea; 4 Department of Nano Science and Technology, Sungkyunkwan University, Suwon-si, Gyeonggi-do 16419 Republic of Korea; 5 Department of Semiconductor Convergence Engineering, Sungkyunkwan University, Suwon-si, Gyeonggi-do 16419 Republic of Korea; 6 AVP Process Development Team, Samsung Electronics, Chungcheongnam-do Cheonan-si, 31086 Republic of Korea; 7 Department of Nano Engineering, Sungkyunkwan University, Suwon-si, Gyeonggi-do 16419 Republic of Korea; 8 Department of Physics, Ewha Womans University, Seoul, 03760 Republic of Korea

**Keywords:** Two-dimensional materials, Mechanical engineering

Correction to: *Nature Communications* 10.1038/s41467-024-48810-3, published online 18 June 2024

In this article Jinhyoung Lee and Gunhoo Woo were incorrectly denoted as corresponding authors. And in this article the peer reviewer in the ‘Peer review information’ was incorrectly given as Hugo Aramberriand but should have been Hugo Aramberri. The original article has been corrected.

