# Supplementary material for: Free-standing two-dimensional ferro-ionic memristor
Source: Nat Commun. 2024 Jun 18;15:5162. doi: 10.1038/s41467-024-48810-3 (PMC11189491; doi:10.1038/s41467-024-48810-3)
Supplement: Supplementary file 1 — Supplementary Information [file 41467_2024_48810_MOESM1_ESM.pdf]

Supplementary Information

Free-standing two-dimensional ferro-ionic memristor

Jinhyoung Lee[1,2,†], *Gunhoo Woo*[3,4,†], *Jinill Cho*[1], *Sihoon Son*[3,4], *Hyelim Shin*[5], *Hyunho Seok*[3,4], *Min-Jae Kim*[3,4], *Eungchul Kim*[6], *Ziyang Wang*[1], *Boseok Kang*[3,4,7], *Won-jun Jang*[2,8] *and Taesung Kim*[*,1,3,4,5,7]

[1]School of Mechanical Engineering, Sungkyunkwan University (SKKU), Suwon-si, Gyeonggi-do 16419, Republic of Korea

[2]Center for Quantum Nanoscience, Institute for Basic Science (IBS), Seoul 03760

[3]SKKU Advanced Institute of Nanotechnology (SAINT), Sungkyunkwan University, Suwon-si, Gyeonggi-do 16419, Republic of Korea

[4]Department of Nano Science and Technology, Sungkyunkwan University, Suwon-si, Gyeonggi-do 16419, Republic of Korea

[5]Department of Semiconductor Convergence Engineering, Sungkyunkwan University, Suwon-si, Gyeonggi-do 16419, Republic of Korea

[6]AVP process development team, Samsung Electronics, Chungcheongnam-do, Cheonan-si 31086, Republic of Korea

[7]Department of Nano Engineering, Sungkyunkwan University, Suwon-si, Gyeonggi-do 16419, Republic of Korea

[8]Department of Physics, Ewha Womans University, Seoul 03760, Republic of Korea

[†] These authors contributed equally to this work.

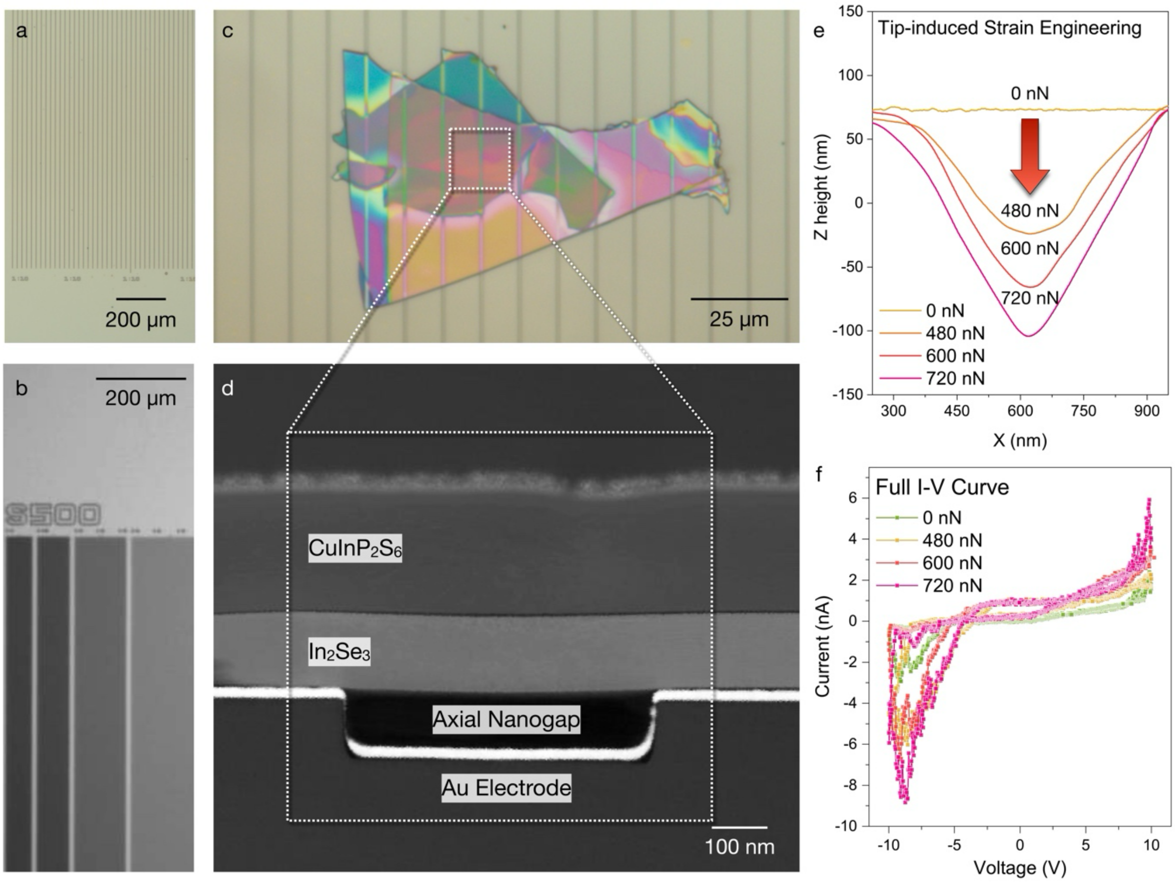

Supplementary Fig. 1. Structural characterization of axial nanogap structure. (a) Optical image of the axial nanogap structure, consisting of 500 nm width and 150 nm depth. (b) SEM image of fabricated axial nanogap array. (c) Optical image of free-standing 2D $\boldsymbol{\alpha}$-$In_2Se_3/CuInP_2S_6$ heterojunction on the axial nanogap structure and (d) its corresponding cross-sectional TEM image of free-standing 2D $\boldsymbol{\alpha}$-$In_2Se_3/CuInP_2S_6$ heterojunction. (e) Line profile and (f) its corresponding CAFM IV curve at the free-standing 2D ferroelectric heterojunction with various applied force, enabling the comparison with free-standing states (0 nN).

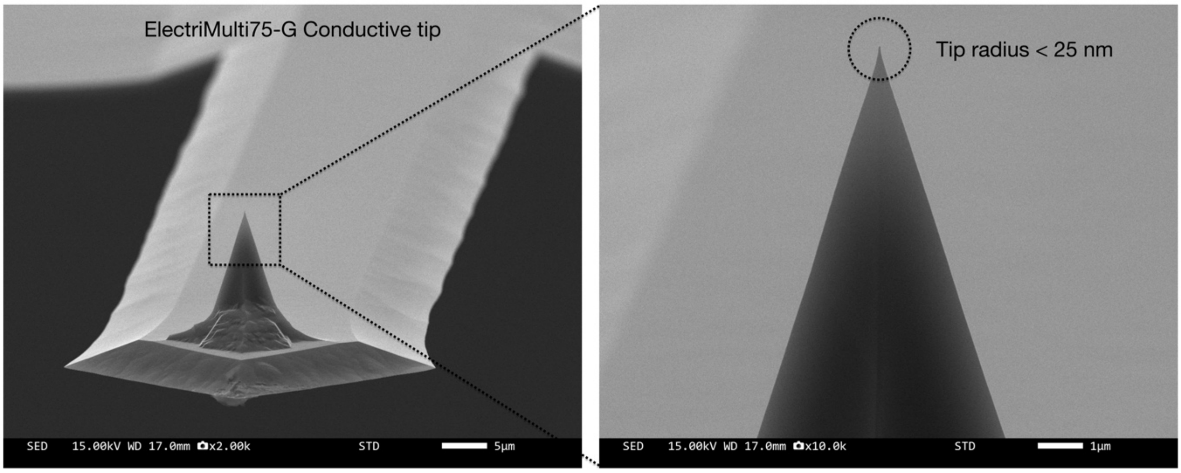

Supplementary Fig. 2. Microscale observation of Platinum conductive AFM tip. SEM image of ElectriMulti75-G tip, which consisted with tip radius 25 nm.

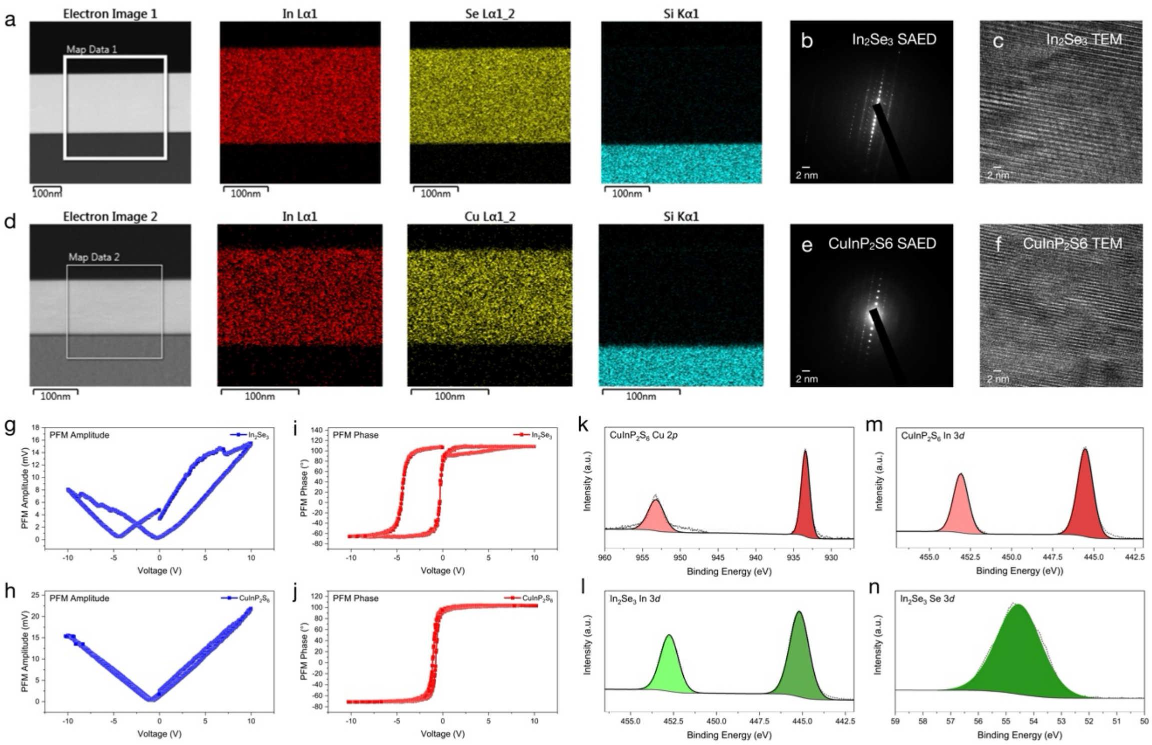

Supplementary Fig. 3. Pre-characterization of cross-sectional atomic configuration and piezoelectric response in single $CuInP_2S_6$/α-In_2Se_3 flake. (a) Cross-sectional EDS mapping, (b) SAED pattern, and (c) cross-sectional TEM image of single α-In_2Se_3 flake. (d) Cross-sectional EDS mapping, (e) SAED pattern, and (f) cross-sectional TEM image of single $CuInP_2S_6$ flake. (g) PFM amplitude of α-In_2Se_3 single flake, (h) PFM amplitude of $CuInP_2S_6$ single flake, (i) PFM phase of α-In_2Se_3 single flake, and (j) PFM phase of $CuInP_2S_6$ single flake. High-resolution XPS spectra of single flake, which can be deconvoluted as (k) Cu 2p peak from $CuInP_2S_6$ and (l) In 3d peak from α-In_2Se_3, (m) In 3d peak from $CuInP_2S_6$, and (n) Se 3d peak from α-In_2Se_3.

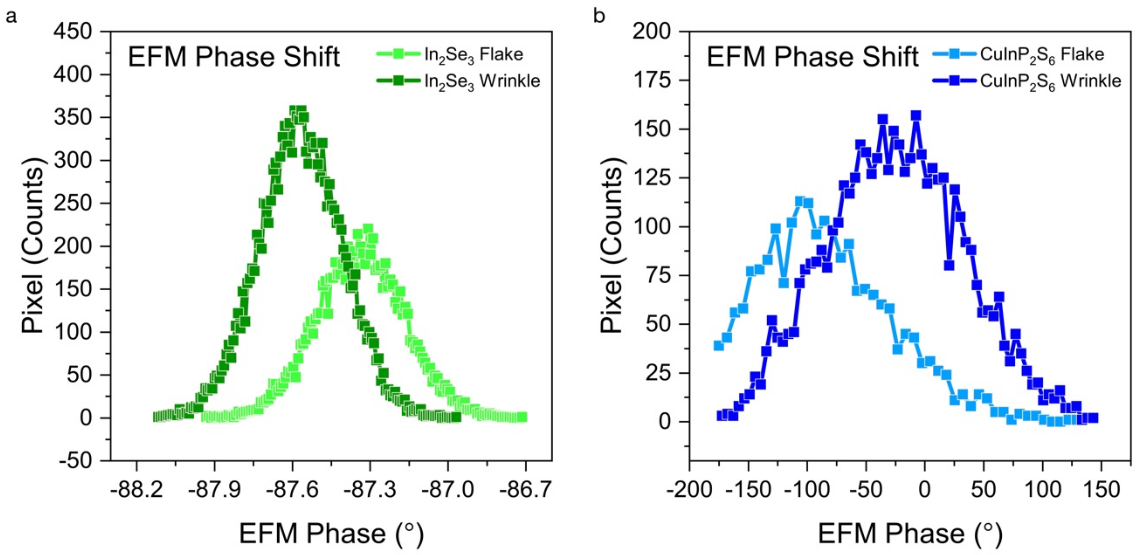

Supplementary Fig. 4. Heterogeneous EFM phase behavior at the wrinkle area. (a) EFM phase shift of wrinkle area and pristine area of α-In_2Se_3 single flake. (b) EFM phase shift of wrinkle area and pristine area of $CuInP_2S_6$ single flake.

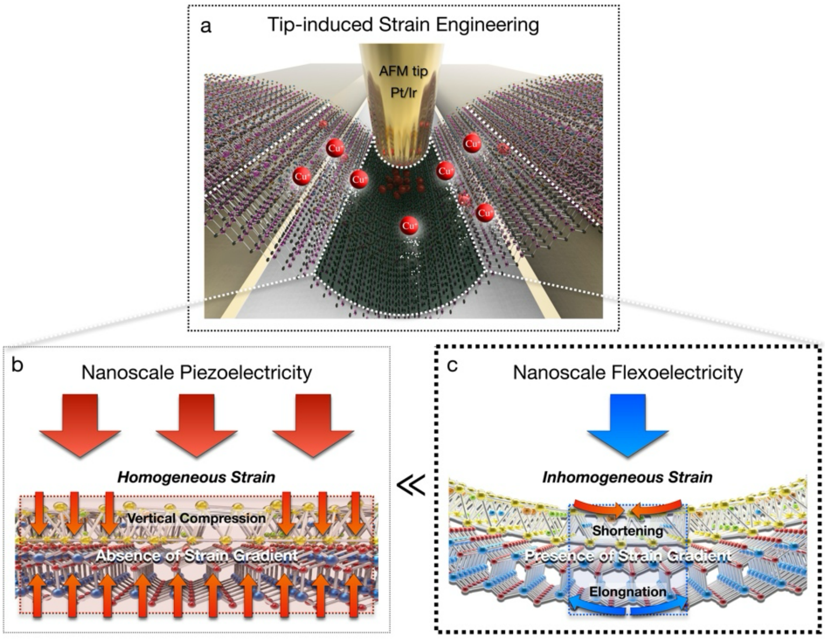

Supplementary Fig. 5. Schematic illustration of piezoelectricity and flexoelectricity of free-standing 2D ferroelectric heterostructure. Schematic of (a) tip-induced strain engineering. Spatial strain distribution of (b) piezoelectricity and (c) flexoelectricity, which is induced with homogeneous strain (piezoelectricity) and inhomogeneous strain (flexoelectricity).

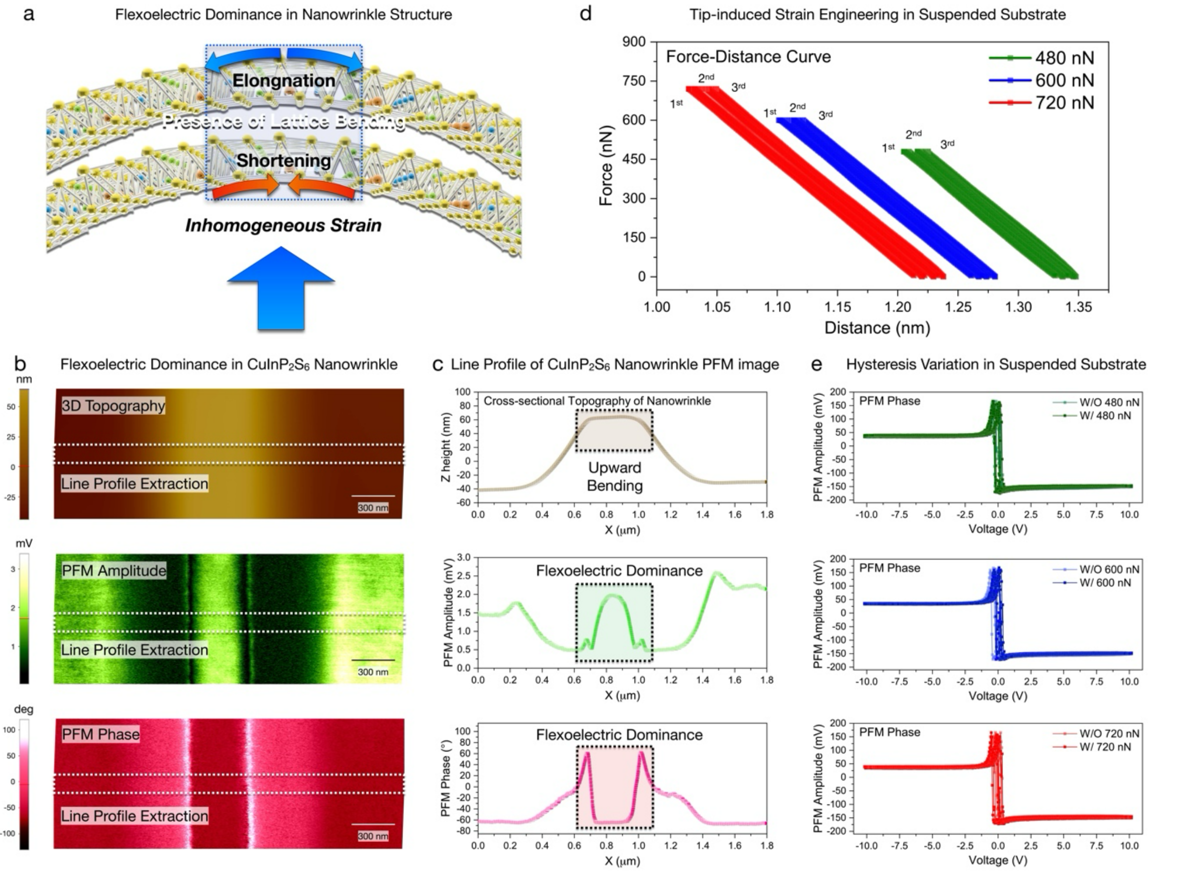

Supplementary Fig. 6. Observation of flexoelectric dominance with nanoscale mechanical bending. Schematic of (a) upward lattice bending in nanowrinkle structure. Spatial distribution of (b) PFM responses and (c) its corresponding line profile, which exhibits the flexoelectric dominance in wrinkle apex area. (d) FD curve measurements at the suspended substrate. (e) PFM hysteresis mapping of substrate suspension, which effectively hinders the lattice bending and its corresponding hysteresis variation with each applied force.

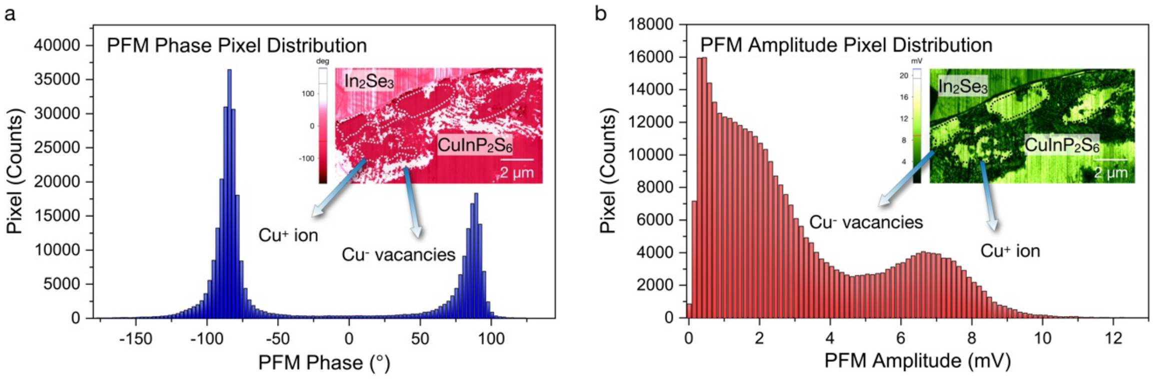

Supplementary Fig. 7. Statical ferroelectric domain mapping with pixel distribution. (a) PFM amplitude and (b) PFM phase, exhibiting randomly distributed Cu^+ ions in the junction area, according to ferroelectric amplification within α-In_2Se_3 polarization and intrinsic Cu^+-Se^{2-} dipole formation.

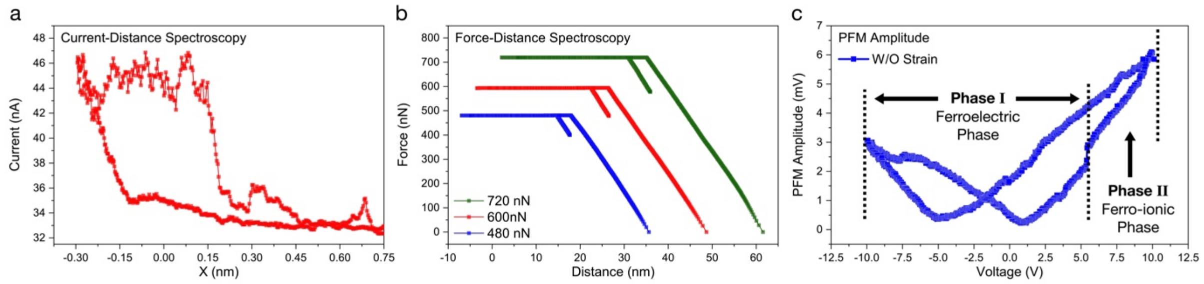

Supplementary Fig. 8. Correlation of nanoscale mechanical bending and flexoelectric ferro-ionic current. (a) current-distance curve and (b) force-distance curve of free-standing 2D $\boldsymbol{\alpha}$-In_2Se_3/$CuInP_2S_6$ heterostructure. (c) PFM amplitude exhibits the ferro-ionic phase without nanoscale mechanical bending, while the paraelectric phase occurred within nanoscale mechanical bending.

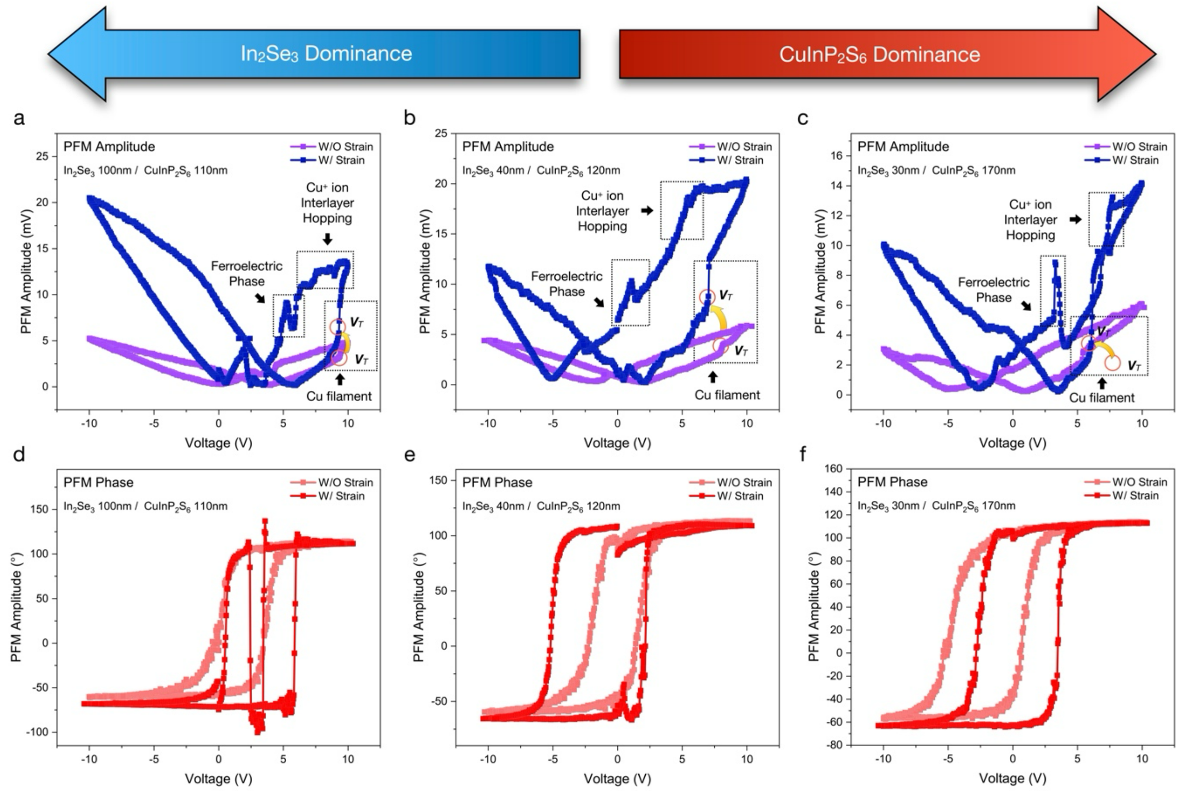

Supplementary Fig. 9. Reversible paraelectric threshold voltage shifts within thickness-dependent layer dominance. PFM amplitude of 2D ferroelectric heterostructure, consisted of (a) ***α***-In_2Se_3 120.70 nm / $CuInP_2S_6$ 115.65 nm (b) ***α***-In_2Se_3 42.94 nm and $CuInP_2S_6$ 151.2 nm (c) ***α***-In_2Se_3 33.36 nm and $CuInP_2S_6$ 180.37 nm. PFM phase of 2D ferroelectric heterostructure, consisted of (d) ***α***-In_2Se_3 120.70 nm / $CuInP_2S_6$ 115.65 nm (e) ***α***-In_2Se_3 42.94 nm and $CuInP_2S_6$ 151.2 nm (f) ***α***-In_2Se_3 33.36 nm and $CuInP_2S_6$ 180.37 nm.

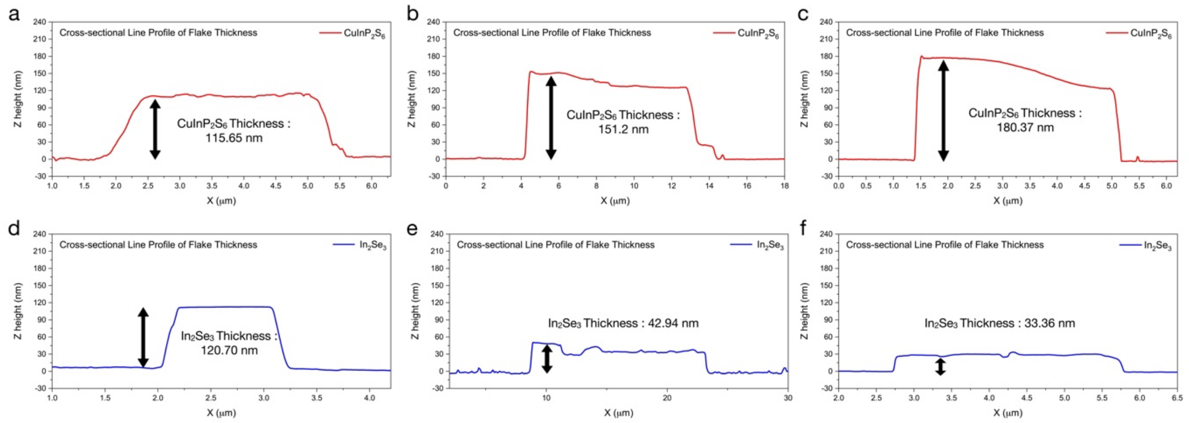

Supplementary Fig. 10. Line profile of each single $CuInP_2S_6$/α-In_2Se_3 flake. (a) $CuInP_2S_6$ thickness 115.65 nm (b) $CuInP_2S_6$ thickness 151.2 nm (c) $CuInP_2S_6$ thickness 180.37 nm (d) α-In_2Se_3 thickness 120.70 nm (e) α-In_2Se_3 thickness 42.94 nm (f) α-In_2Se_3 thickness 33.36 nm.

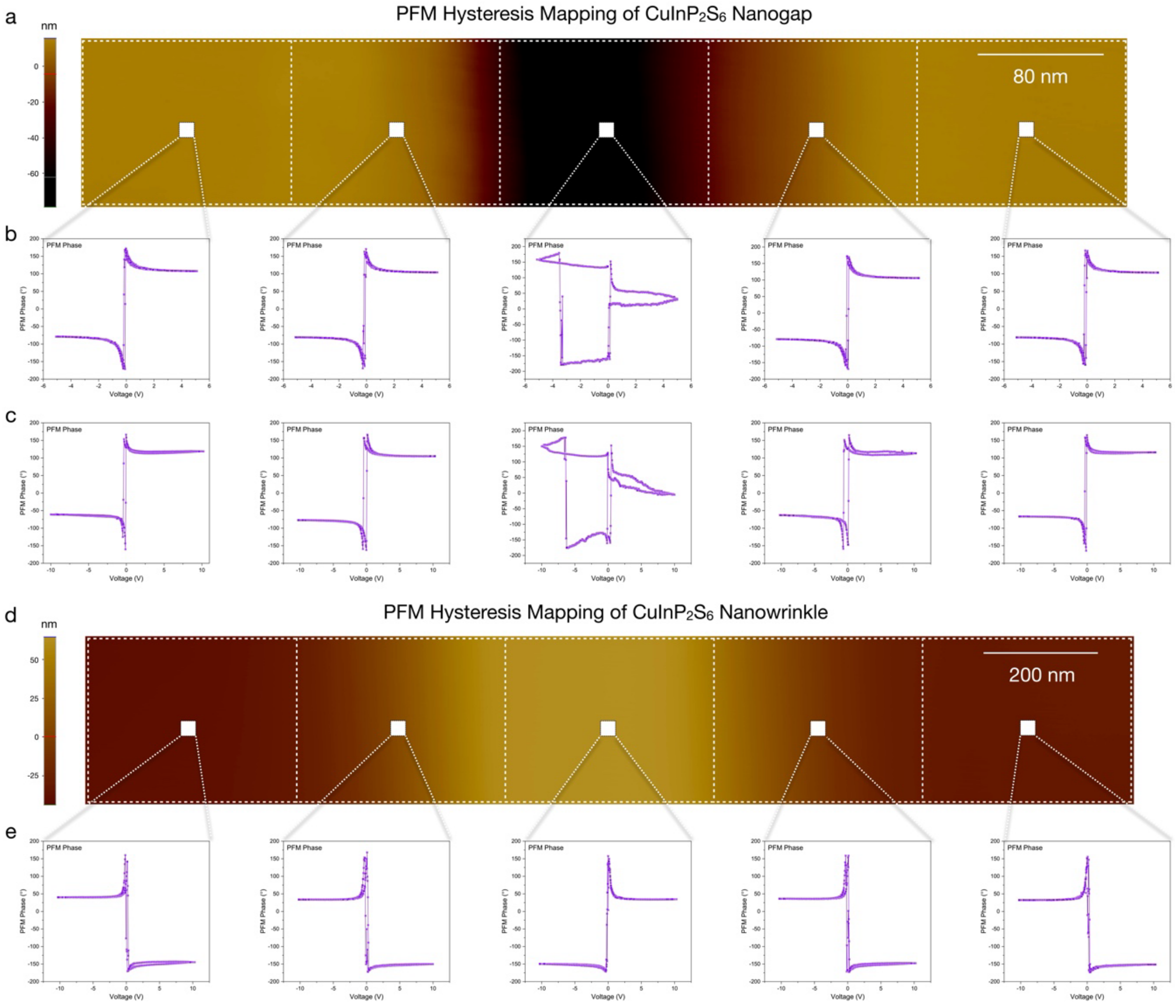

Supplementary Fig. 11. Spatial PFM hysteresis mapping of nanoscale axial bending structure. (a) 3D topography of axial nanogap structure and PFM hysteresis mapping within (b) -5 V–5 V sweep range (ferro-ionic phase) and (c) -10 V–10 V sweep range (paraelectric phase). (d) 3D topography of axial nanowrinkle structure and PFM hysteresis mapping within (e) -10 V–10 V sweep range.

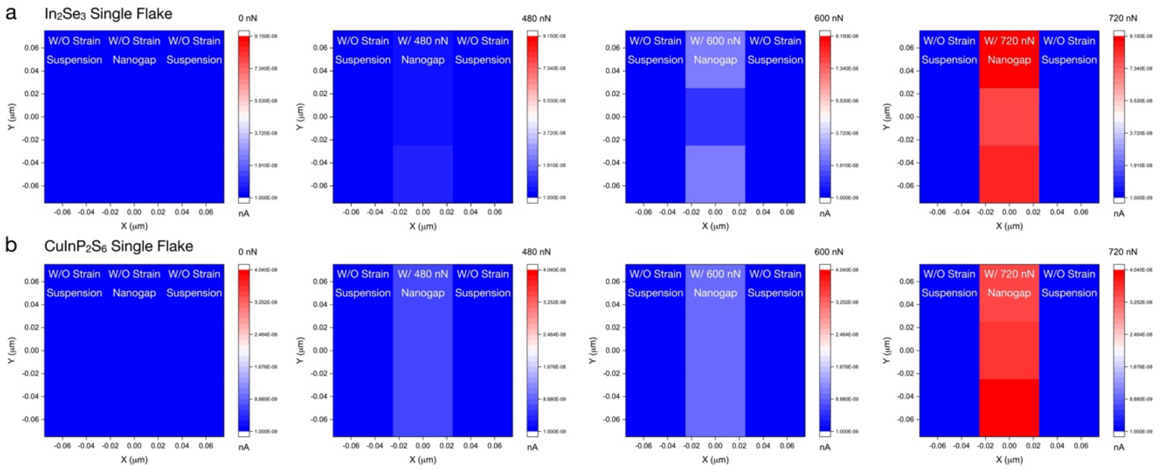

Supplementary Fig. 12. Spatial I_{max} distribution of single $CuInP_2S_6$/$\boldsymbol{\alpha}$-In_2Se_3 flake within localized flexoelectric current amplification. Spatial I_{max} distribution of (a) single α-In_2Se_3 memristor and (b) single $CuInP_2S_6$ memristor, which indicates the controllable ferro-ionic conduction within sub-50 nm resolution.

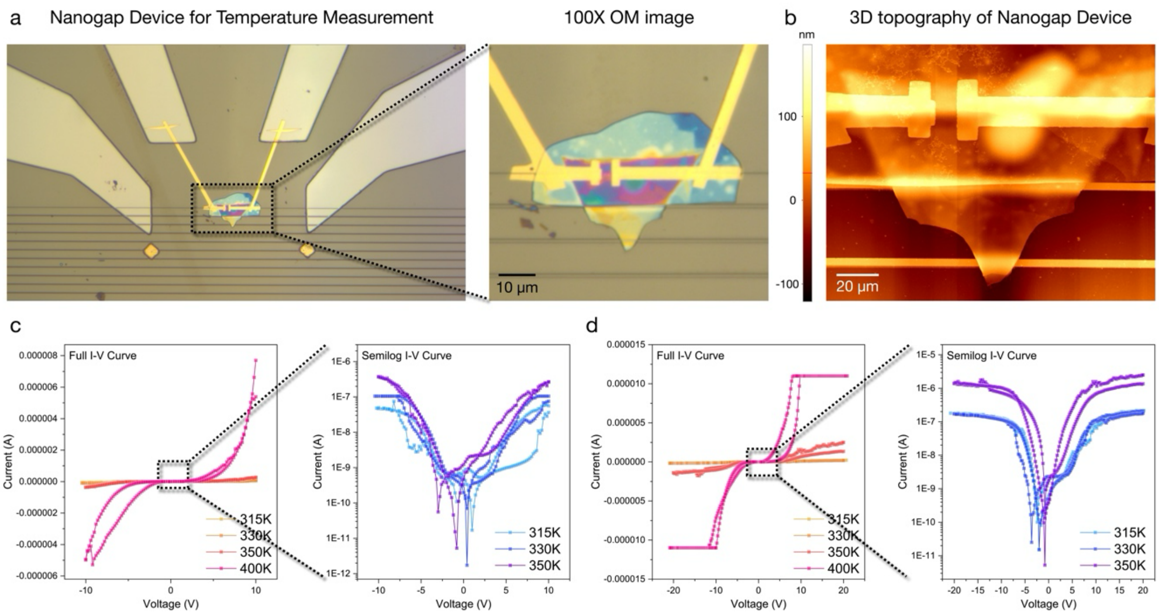

Supplementary Fig. 13. Temperature effects in free-standing 2D ferroelectric nanogap device. (a) OM image and (b) 3D topography of free-standing 2D nanogap device, which is fabricated for temperature effects. Within various temperature conditions, ferro-ionic conduction mapping at (c) -10 V–10 V sweep range, and (d) -20 V–20 V sweep range, exhibiting the temperature-independent conduction in 315K, 330K, and 350K.

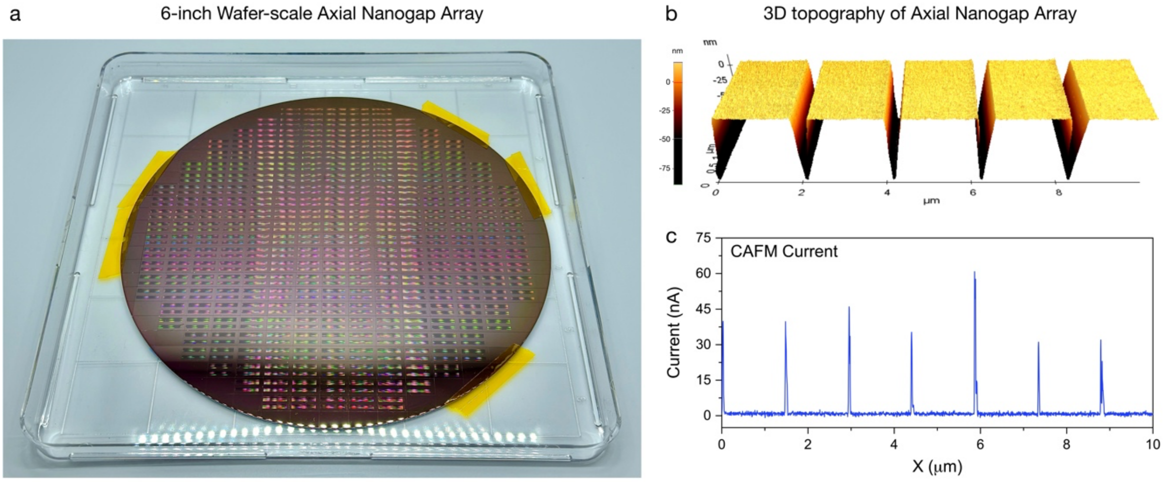

Supplementary Fig. 14. Geometric extendibility of wafer-scale axial nanogap array. (a) Photography of 6-inch wafer-scale axial nanogap array. (b) 3D topography and (c) its corresponding CAFM current mapping at the axial nanogap array.

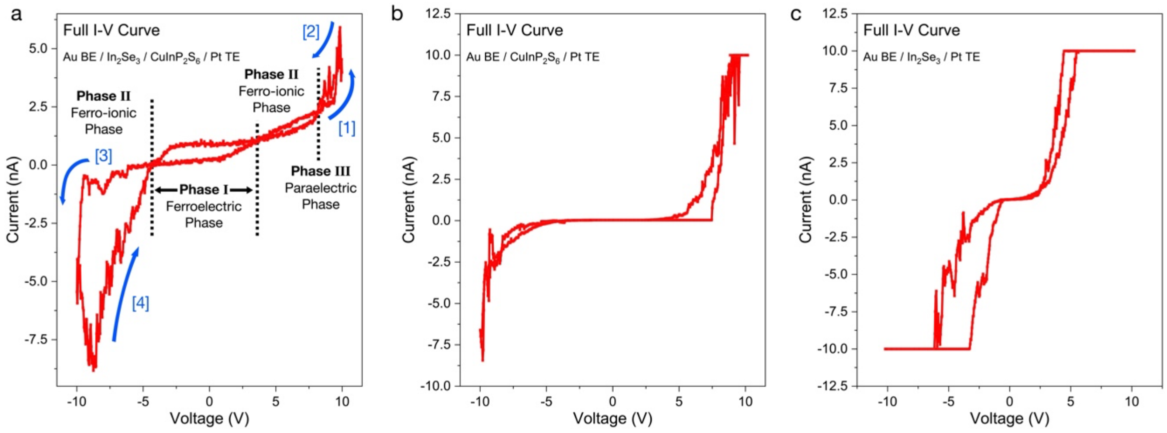

Supplementary Fig. 15. Full I-V curve measurement of single 2D memristive behavior. Full I-V curve of (a) free-standing 2D hetero-stacked memristor device, consisted of Au bottom electrode/$\boldsymbol{\alpha}$-In_2Se_3/$CuInP_2S_6$/Pt top electrode, (b) Au bottom electrode/$CuInP_2S_6$/Pt top electrode, and (c) Au bottom electrode/$\boldsymbol{\alpha}$-In_2Se_3/Pt top electrode.

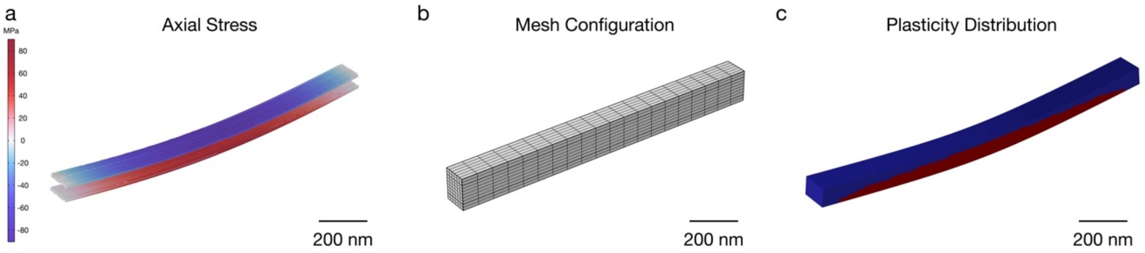

Supplementary Fig. 16. Simulation condition for theoretical flexoelectric calculation. 3D simulation and its corresponding simulation condition, which is consisted of (a) axial stress, (b) simulation mesh configuration, and (c) plasticity distribution within experimentally obtained mechanical bending curvature (480 nN, 600 nN, 720 nN) at the free-standing states.

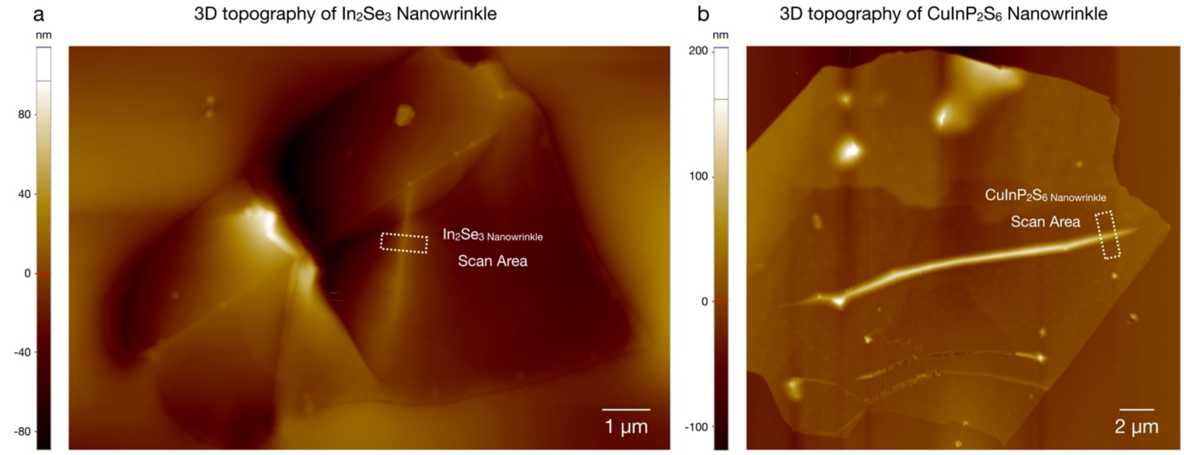

Supplementary Fig. 17. 3D topography of nanowrinkle structure in transferred 2D ferroelectric materials. Spatial topography image of transferred (a) nanowrinkle $CuInP_2S_6$, (b) nanowrinkle In_2Se_3.
