# Supplementary material for: Free-standing two-dimensional ferro-ionic memristor
Source: Nat Commun. 2024 Jun 18;15:5162. doi: 10.1038/s41467-024-48810-3 (PMC11189491; doi:10.1038/s41467-024-48810-3)
Supplement: Supplementary file 3 — Reporting Summary [file 41467_2024_48810_MOESM3_ESM.pdf]

nature portfolio

Corresponding author(s): Taesung Kim

Last updated by author(s): May 12, 2024

Reporting Summary

Nature Portfolio wishes to improve the reproducibility of the work that we publish. This form provides structure for consistency and transparency in reporting. For further information on Nature Portfolio policies, see our Editorial Policies and the Editorial Policy Checklist.

Statistics

For all statistical analyses, confirm that the following items are present in the figure legend, table legend, main text, or Methods section.

n/a	Confirmed	
☐	☒	The exact sample size (*n*) for each experimental group/condition, given as a discrete number and unit of measurement
☐	☒	A statement on whether measurements were taken from distinct samples or whether the same sample was measured repeatedly
☒	☐	The statistical test(s) used AND whether they are one- or two-sided<br>*Only common tests should be described solely by name; describe more complex techniques in the Methods section.*
☒	☐	A description of all covariates tested
☒	☐	A description of any assumptions or corrections, such as tests of normality and adjustment for multiple comparisons
☐	☒	A full description of the statistical parameters including central tendency (e.g. means) or other basic estimates (e.g. regression coefficient) AND variation (e.g. standard deviation) or associated estimates of uncertainty (e.g. confidence intervals)
☐	☒	For null hypothesis testing, the test statistic (e.g. *F*, *t*, *r*) with confidence intervals, effect sizes, degrees of freedom and *P* value noted<br>*Give P values as exact values whenever suitable.*
☒	☐	For Bayesian analysis, information on the choice of priors and Markov chain Monte Carlo settings
☒	☐	For hierarchical and complex designs, identification of the appropriate level for tests and full reporting of outcomes
☒	☐	Estimates of effect sizes (e.g. Cohen's *d*, Pearson's *r*), indicating how they were calculated

Our web collection on statistics for biologists contains articles on many of the points above.

Software and code

Policy information about availability of computer code

Data collection	N/A
Data analysis	N/A

For manuscripts utilizing custom algorithms or software that are central to the research but not yet described in published literature, software must be made available to editors and reviewers. We strongly encourage code deposition in a community repository (e.g. GitHub). See the Nature Portfolio guidelines for submitting code & software for further information.

Data

Policy information about availability of data

All manuscripts must include a data availability statement. This statement should provide the following information, where applicable:

- Accession codes, unique identifiers, or web links for publicly available datasets
- A description of any restrictions on data availability
- For clinical datasets or third party data, please ensure that the statement adheres to our policy

The source data generated in this study are provided in the Supplementary Information/Source Data file. All other data that support this study are available from the corresponding authors upon reasonable request.

Research involving human participants, their data, or biological material

Policy information about studies with human participants or human data. See also policy information about sex, gender (identity/presentation), and sexual orientation and race, ethnicity and racism.

Reporting on sex and gender	N/A
Reporting on race, ethnicity, or other socially relevant groupings	N/A
Population characteristics	N/A
Recruitment	N/A
Ethics oversight	N/A

Note that full information on the approval of the study protocol must also be provided in the manuscript.

Field-specific reporting

Please select the one below that is the best fit for your research. If you are not sure, read the appropriate sections before making your selection.

☐ Life sciences ☐ Behavioural & social sciences ☒ Ecological, evolutionary & environmental sciences

For a reference copy of the document with all sections, see nature.com/documents/nr-reporting-summary-flat.pdf

Ecological, evolutionary & environmental sciences study design

All studies must disclose on these points even when the disclosure is negative.

Study description	Enhanced ferroelectric characteristics in CIP2S6 have been confirmed via nano-scale flexoelectric and In2Se3 poling effect and implemented improved ferro-ionic memristor characteristics. We prepared CIP2S6/In2Se3 heterostructure and controlled various parameters such as the bending radius, materials thickness, and temperature to explore the ferro-ionic characteristics.
Research sample	We have utilized CuInP2S6 and In2Se3 ferroelectric insulator and semiconductor, which are representative 2D materials that possess unique polarization controllability according to external force, bending, and bias.
Sampling strategy	To prepare the CIP2S6/In2Se3 heterostructure, we employed the most representative dry transfer process. From the bulk materials, thin CIP2S6 and In2Se3 were exfoliated and stacked via the dry transfer stage using a PDMS supporting layer.
Data collection	Jinhyoung Lee and Gunhoo Woo prepared the data set using AFM equipment and Keithley equipment to investigate materials characteristics and memristor performance. The material condition was checked with materials characteristics analysis tools like XPS, TEM, and FE-SEM.
Timing and spatial scale	The data collection had taken for 2 months, which started from 10/6 to 12/26. Most of the main data was obtained within 2 days, and additional data was collected with 1 week period. Since we possessed our own AFM and Keithley equipment and the sampling preparation procedure was relatively simple, the overall sampling process was conducted speedily.
Data exclusions	Every data from each sample has been utilized in the paper.
Reproducibility	To confirm the reproducibility of our findings, we evaluated the flexoelectric effect at 3 different nanogap positions for each tip-induced strain condition. Every result shows an identical data tendency.
Randomization	We had comprised a single sample group according to the width/depth of the nanogap and the thickness of the CIP2S6/In2Se3.
Blinding	There is no blinding in our research. All data are plotted using raw data.

Did the study involve field work? ☐ Yes ☒ No

Reporting for specific materials, systems and methods

We require information from authors about some types of materials, experimental systems and methods used in many studies. Here, indicate whether each material, system or method listed is relevant to your study. If you are not sure if a list item applies to your research, read the appropriate section before selecting a response.

Materials & experimental systems

n/a	Involved in the study
☒	☐ Antibodies
☒	☐ Eukaryotic cell lines
☒	☐ Palaeontology and archaeology
☒	☐ Animals and other organisms
☒	☐ Clinical data
☒	☐ Dual use research of concern
☒	☐ Plants

Methods

n/a	Involved in the study
☒	☐ ChIP-seq
☒	☐ Flow cytometry
☒	☐ MRI-based neuroimaging

Plants

Seed stocks	N/A
Novel plant genotypes	N/A
Authentication	N/A
